# Review of the Lithuanian Alcohol Control Legislation in 1990–2020

**DOI:** 10.3390/ijerph17103454

**Published:** 2020-05-15

**Authors:** Laura Miščikienė, Nijolė Goštautaitė Midttun, Lukas Galkus, Gražina Belian, Janina Petkevičienė, Justina Vaitkevičiūtė, Mindaugas Štelemėkas

**Affiliations:** 1 Health Research Institute, Faculty of Public Health, Lithuanian University of Health Sciences, 44307 Kaunas, Lithuania; laura.miscikiene@lsmuni.lt (L.M.); lukas.galkus@lsmuni.lt (L.G.); janina.petkeviciene@lsmuni.lt (J.P.); justina.vaitkeviciute@lsmuni.lt (J.V.); 2 Department of Health Management, Faculty of Public Health, Lithuanian University of Health Sciences, 44307 Kaunas, Lithuania; 3 Lithuanian Tobacco and Alcohol Control Coalition, LT-01131 Vilnius, Lithuania; nigomi@hotmail.com; 4 Mental Health Initiative, LT-03107 Vilnius, Lithuania; 5 Drug, Tobacco and Alcohol Control Department, LT-01312 Vilnius, Lithuania; grazina.belian@ntakd.lt; 6 Department of Preventive Medicine, Faculty of Public Health, Lithuanian University of Health Sciences, 44307 Kaunas, Lithuania

**Keywords:** alcohol, alcohol policy, policy process, legislation

## Abstract

Since the early 1990s, Lithuania has experienced an increasing level of alcohol consumption and a heavy burden of alcohol-related harm, which is associated with the development of alcohol policies. The aim of this analysis was to provide a chronology of change of Lithuanian alcohol control legislation and to present several other detailed examples of the political processes. The data were collected using document reviews. During the last three decades, the Lithuanian alcohol control policies have undergone several cycles of stricter control and liberalizations. Some of the limitations of the study are the exceptional focus on the public health perspective and the inclusion of policies targeting the population as a whole. The strength of the study is in providing a detailed background for future policy effectiveness studies. Some of the recent periods when a series of ‘best buy’ interventions were implemented during a short period are of particular importance, constituting a natural experiment, whose effects need to be studied in more detail in the future.

## 1. Introduction

Alcohol burden has been a challenge to public health in many countries around the world, and especially in Europe for the last couple of decades. In 2006, the EU adopted a strategy to support the Member States in reducing alcohol-related harm, and the need for a policy response was re-affirmed in 2008 and 2010 in a consensus call to develop a global strategy to reduce the harmful use of alcohol at the World Health Assembly [1]. Nonetheless, the adoption of policies outlined in various international documents has proven to be an arduous and slow process at the national level [2,3]. 

The history of the Lithuanian alcohol policy development is no exception. Lithuania is a European Union (EU) member state on the Baltic Sea with a population of 2.8 million and is ranked as a full democracy [4]. It is a high-income country and a member of the Economic Co-operation and Development Organization (OECD) and the North Atlantic Treaty Organization (NATO), memberships that are strongly supported by the population [5,6]. 

In 2020, Lithuania is notable not only as one of the countries with the highest alcohol consumption, but also for its demonstrable evidence of the public health benefits associated with alcohol control policy. The World Health Organization (WHO) indicates that alcohol consumption in 2016 was 15 liters per capita (15+) [1], which translated into significant consequences for public health. In 2016, alcohol was implicated in approximately 7.6% of total deaths in Lithuania, equivalent to nearly 3100 deaths in 2016 alone [7]. 

The burden of alcohol-related social harm is reflected in many other indicators. In 2016, 57% of murders, 48% of severe violent injuries, and 42% of rapes were committed under the influence of alcohol. Links to alcohol consumption were identified in six out of 10 cases of domestic violence and nine out of 10 cases, when children had to be removed from families and placed under child protection in foster care or institutions. Economic costs related to alcohol in 2016 constituted approximately 325 million euros or 0.85% of GDP [7]. 

Consumption indicators and policies aimed at reducing consumption have faced dramatic changes during the three decades after the country regained its independence in 1990. Lithuania emerged from Soviet occupation with relatively low consumption due to a very restrictive Union of Soviet Socialist Republics (USSR) wide anti-alcohol campaign launched in 1985. The reform was short-lived, but had a significant positive public health impact on the three Baltic states (Lithuania, Latvia, and Estonia) as well as in Russia, which was exemplified by a rise in life expectancy lasting until 1987, then followed by a similar trend of decline in the early 1990s [8]. The average USSR consumption of state produced alcohol in 1990 was estimated at 5.56 liters per capita [9], which stands in stark contrast to a peak consumption in Lithuania in 2010 at 15.4 liters of pure alcohol per capita (15+ years population) [10]. 

The Declaration of Independence of the Republic of Lithuania restored the pre-occupation Constitution of 1938, and declared illegitimacy of any foreign constitution and laws from 11 March 1990 [11]. This effectively created a situation in which nearly all state regulation of any aspects of production, import, and sale of alcohol was disabled until the introduction of the Alcohol Control Law in 1995. Missing legislation, poor border control, and significant personal financial gains from alcohol sales resulted in a dramatic rise in alcohol consumption in the first half of the 1990s. Alcohol consumption stabilized after the Alcohol Control Law was adopted and relevant control institutions were established in 1995. Thus, implementation of alcohol control policy in Lithuania commenced undergoing both stricter control and liberalization cycles. 

The aim of this study was to provide an overview of the legal acts constituting Lithuanian alcohol policy and legislative processes underpinning their adoption. This would contribute to internationally applicable evidence for future research analysis of the impact of alcohol control policies on social and public health indicators. The chronological evolution of relevant changes from 1990 to 2020, and specific case examples provide insights into the process of policy development in a democratic political legislative context.

## 2. Materials and Methods 

### 2.1. Data Collection and Study Design

The definition of alcohol control policy is based on the rationale and principles outlined in the WHO indicator “adopted written national policy on alcohol”: a written, organized set of values, principles, and objectives for reducing the burden attributable to alcohol in a population, which is adopted at the national level [1]. The analysis included changes in laws and other regulations adopted by the highest policy-making authorities in the country, the Seimas of the Republic of Lithuania (hereinafter referred to as the Parliament) and the Government of the Republic of Lithuania (hereinafter referred to as the Government). 

The WHO “Global strategy to reduce the harmful use of alcohol” adopted by consensus resolution WHA 63.13 in May 2010 by the World Health Assembly was used to identify evidence-based alcohol policy actions recommended at the national level. The authors selected five policy actions (out of 10 listed) with direct and significant impact on the population alcohol consumption: leadership, awareness and commitment; drink-driving policies and countermeasures; availability of alcohol; marketing of alcoholic beverages; and pricing policies. These were labelled as five control policy categories: general policy, taxation, availability, marketing, and drink-driving. The study authors reviewed and selected laws and other legal documents matching the five control policy categories above-mentioned. Six legal acts were identified for the analysis: Law on Alcohol Control, the Resolution on Excise Duty, the Law on Excise Duty, the Code of Administrative Offenses, the Criminal Code, and the Resolution on Licensing Procedure for the Production of Alcohol Products. 

In the next stage, all amendments (281 in total) to the selected legal acts adopted from 1990 to 2020 were obtained from the Register of Legal Acts [12], out of which 63 were amendments to the Law on Alcohol Control, 51 to the Law on Excise Duty, 20 to the Resolution on Excise Duties, 52 to the Code of Administrative Offenses, 71 to the Criminal Code, and 24 to the Resolution on Licensing Procedure for the Production of Alcohol Products. 

Two researchers separately read and analyzed the content of all amendments and reported to the authors’ group. Amendments then were analyzed by the group regarding their potential impact on the population alcohol consumption, date of entry into force, administrative regulation, and 46 amendments pertaining to alcohol control and the five identified categories were selected for the analysis by consensus. In addition, the group identified and included three national public health programs (National Health Program 1998–2010; National Health Program 2014–2025; National Program for Drug, Tobacco and Alcohol Control and Prevention 2018–2028) and one additional policy document declaring 2008 as The Year of Sobriety into the analysis. Thus, 52 timeline points were included in the study. The analysis was performed in February and March 2020.

### 2.2. Case Examples 

The aim of the case examples was to provide details of the process of policy development. The specific cases were chosen based on a high level of publicity and controversy in society at the time. In total, four such examples were identified, one of which has already been published as a separate case study of a failed attempt to introduce an alcohol advertising ban from 2012 [13], and was therefore excluded. 

Three timeline related examples are demonstrated in the article:

1) Alcohol sale ban in petrol stations which came into effect in 2016;
2) A brief industry campaign of universal personal ID verification for buying alcohol in 2016; and
3) Journal page ripping initiative: a response to the advertising ban in 2018.

The qualitative descriptions were based on Internet media article coverage, press releases, and data collected by the Lithuanian Tobacco and Alcohol Control Coalition at the time of events including the chronology of the alcohol sales ban in petrol stations from 2011 to 2015 [14].

## 3. Results

The Lithuanian alcohol control policy is based on laws, which provide policy targets, define and describe the policy measures and framework of implementation. All strategic policy documents are adopted through the democratic legislative process either as laws or as resolutions by the Parliament and the Government, respectively. The Law on Alcohol Control serves as the main policy document, supplemented by some general policies and other laws. 

The main policy document has the reputation of being the most frequently amended act in Lithuanian legislative history. Since 1995, a total of 63 amendments have come into effect [15]. 

### 3.1. Timeline of Alcohol Control Legislation

**Figure 1:**
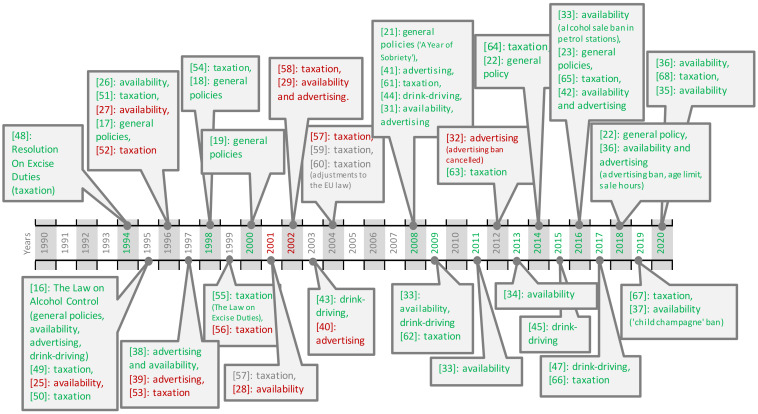
Timeline of Lithuanian alcohol control policies listed up by the policy category. Note: numbers in the figure reflect the listed reference numbers; green color indicates stricter policy measures; red—liberalization; grey—uncertain/difficult to attribute.

There are 52 entries identified in the timeline of the Lithuanian alcohol control policy processes, which are listed by the date of the legislation coming into effect. Some of the timeline points cover different policy categories implemented with the same amendment. Figure 1 provides a summary of the timeline events with relevant dates. The results are displayed in five policy categories: general policies, taxation, availability, marketing, and drink-driving policy measures.

#### 3.1.1. General Policies 

There are eight timeline points in the first category of general policies that reflect the targeted political commitment toward alcohol control. This category had a major positive impact on public health during the early development of the alcohol control policy and was most actively implemented between 1995 and 2000. The Law on Alcohol Control [16], adopted after more than two years of deliberations by the Parliament and came into effect on 26 May 1995, created the foundations for the policy. It is the first and most highly significant policy document since it outlines the purpose “to reduce consumption of alcohol, availability, especially to minors, alcohol abuse, the damage caused by it to health and the economy and to establish legal principles of granting economic entities the right to manufacture, sell, import and export alcohol products.” 

Initially, policy implementation of the law was delegated to the ministries, and then one year later, from 20 June 1996, to a newly established supervisory body: the State Agency for Tobacco and Alcohol Control [17]. Another general policy document demonstrating leadership and commitment was the Lithuanian Health Strategy [18], which began in 1998. The Strategy established specific targets for the reduction of alcohol consumption, and indicators of morbidity and mortality due to alcohol use for the first time. Then, on 15 March 2000, amendments to the Code of Administrative Offenses came into effect (adopted earlier in the same year) [19], which comprehensively transformed a large number of punitive norms. The changes included regulation of alcohol consumption in public and workplaces, and the introduction of punishments for violations of the alcohol retail rules. It also increased existing administrative fines and penalties for violations. The Code of Administrative Offenses introduced differentiated liability based on the severity of intoxication, classifying it into mild (0.41–1.5‰), medium (1.51–2.5‰), or severe (>2.50‰) intoxication. A new norm criminalizing a repeated drink-driving offence was newly added to the Criminal Code [20], coming into effect in April 2000.

This period set the foundation for all future changes in alcohol policy. Later demonstrations of political will and commitment to alcohol control were either mostly symbolic such as declaring the year 2008 “A Year of Sobriety” [21] or follow up amendments such as the renewed Lithuanian Health Strategy (for the period of 2014–2025) [22]. The next legislation that marks renewed commitment and potentially a new national action category for future analysis was the establishment of the State Fund for Public Health Promotion from 1 January 2016, which created a new significant funding stream for community action toward the prevention of alcohol harm and promotion of public health [23]. Another significant development was the new and comprehensive 2018–2028 National Program for Drug, Tobacco, and Alcohol Control and Prevention [24] adopted at the very end of 2018. The program features a strong commitment to developing a healthcare response, integrates varied policy responses toward all psychoactive substance use, and sets a perspective of policy development for a decade. Most of the specific legal decisions and actions according to this document are planned in the future.

#### 3.1.2. Availability

There are 15 timeline points in the availability category. The Law on Alcohol Control [16], adopted 26 May 1995, laid the foundation for the regulation of the physical availability of alcohol and outlined the rules for alcohol production, control, and sales. The initial version banned the production of home-brewed alcoholic beverages, except naturally fermented beverages below 18% (below 9.5% for beer) for personal use (i.e., not for sale). The law gave specific companies the right to produce undenatured ethyl alcohol and alcoholic beverages, although it simultaneously stated that all companies producing alcoholic beverages and undenatured ethyl alcohol were state-owned. Thus, there was a de facto monopoly on the production of alcohol stronger than 22%. 

Alcohol retail was also restricted. The legal age limit was set to 18 years and sales to intoxicated people and uniformed officers were forbidden. Selling alcoholic beverages in health care, education, sports facilities and their territories, and in front of the prayer buildings (within a distance determined by the municipalities together with religious communities) was banned. There was also a ban on the sale of alcohol in shops selling children’s merchandise, from vending machines, petrol stations, and roadsides. Only sales of alcoholic beverages below 12% strength were permitted during mass sports, cultural and religious festivals, rallies, and demonstrations. In addition, the law provided alternative ways to restrict sales with regulations outside the law.

Alcohol retail was permitted after 10 p.m., but it was subject to double tariffs. By then, municipalities had been granted a right to limit alcohol retail time, but this right was later restricted by the courts. 

Later amendments approved on 26 July 1995 [25] liberalized beer imports, revoked the double tariffs for sales permit on Sundays, extended beer retail time by three hours, and introduced differentiated penalties according to the severity of the violation.

Following the liberalization of alcohol production, the Government adopted a licensing system for the production of alcohol products [26] with defined permit expiry times. Clauses for suspension or cancellation of license in cases of fraud, law violations, inventory discrepancies, etc. were instituted. Other minor amendments facilitated the procedure for wine import and permitted beer retail beyond specialized departments and at other points of sale [27].

Further amendments that came into effect on 28 November 2001 [28] allowed for alcohol retail at petrol stations. The state production monopoly was abolished with the amendment that came into effect on 28 June 2002 [29]. However, it granted municipalities more rights to restrict or prohibit the sale of alcoholic beverages and designate environments where consumption was prohibited. The possibility of state alcohol monopoly was also removed by the Constitutional Court on 26 January 2004 [30]. 

Amendments from 2008 to 2011 focused on strengthening the restrictions on physical availability by limiting sales. The sale of alcoholic beverages (except naturally fermented) with a strength exceeding 13% was banned during mass events [31] and was followed by the ban on the sale of alcoholic beverages in kiosks, sanatoriums, at night (10 p.m. to 8 a.m.) and on 1 September (the formal date of the new academic year in Lithuania). Additionally, persons under the age of 18 were forbidden from possessing and consuming alcoholic beverages and penalties for violations of alcohol control laws, especially for advertising, increased. The right to request a document for age validation from any client was added to the norm of the legal purchasing age. Furthermore, the amendment banned the sale of alcoholic beverages in petrol stations from 2016 onward [32,33].

In 2013, it was prohibited to sell fermented beverages and alcoholic cocktails bottled in packaging over one liter, except for those in glass, ceramic, wooden, or metal packaging, and beer, fermented beverages, alcoholic cocktails below 7.5% of strength bottled in the packaging of over 0.5 liters, except for those in glass, ceramic, wooden, or metal packaging [34].

In 2019, packaging requirements were further restricted. Packaging for alcohol categorized as beer, fermented beverages, and alcoholic cocktails stronger than 6% could no longer be bottled in smaller vessels than 0.2 liters, while those lower than 6% could no longer be bottled in packaging larger than 1 liter. These restrictions did not apply to products bottled in glass, ceramic, wooden, or metal packaging. The amendment also indicated a ban on the sale of alcohol with strengths exceeding 22% in factory glasses and other containers for direct consumption [35].

Several significant policies were adopted in 2017 and came into effect in 2018, 2019, and 2020. Since 1 January 2018, the legal age to purchase and consume alcohol was increased from 18 to 20 years. Retailers were obliged to ask for identification if they were unsure whether the buyer was at least 25 years of age. Retail hours for alcohol sale off-premises were reduced from 10 a.m. until 8 p.m. on Mondays to Saturdays, and from 10 a.m. to 3 p.m. on Sundays (exceptions: airports, ferries, train bars/shops) [36].

Since 1 November 2019, it is prohibited to manufacture and to sell food, toys, and other goods for children and adolescents whose designs mimic alcoholic beverages and/or their packaging (i.e., a ban of champagne for children) [37].

Since 1 January 2020, a ban on alcohol sales on the beaches (seasonal alcohol licenses terminated), in pavilions (more stringent control of night-time off-premise retail), and alcoholic beverages stronger than 7.5% during public events came into effect. Outdoor alcohol retail was only permitted within a 40 meter radius from the vendor building, and expanded rights for municipalities to issue additional restrictions (applicable to catering establishments). A special type of seasonal license to sell alcoholic beverages during the holiday, recreation, and tourism season was abolished [36].

#### 3.1.3. Advertising

There are 10 timeline points in the advertising category. A first full ban on advertising alcoholic beverages was adopted within the initial version of the Law on Alcohol Control [16]. The ban covered locally produced radio and television programs, print media, specialized advertising brochures as well as indirect advertising. However, none of these measures were strictly enforced. The ban was challenged in the Constitutional Court, which on 13 February 1997 ruled that alcoholic beverages and tobacco products are a ‘special purpose goods’ due to the “harm caused by these products to the society”, and therefore market restrictions such as the ban on alcohol advertising would not be unconstitutional [38]. 

When this attempt to remove advertising restrictions failed, a new liberalizing amendment was proposed, adopted, and came into effect on 16 July 1997 [39], with only two weeks from adoption in the Parliament and coming into effect date. This amendment essentially permitted all advertisement of alcohol, while outlining specific prohibited practices such as alcohol advertising targeting children under 18, and false or misleading information about alcoholic beverages. The amendment also detailed what is not permitted in the ad itself: the use of underage persons, athletes, doctors, politicians, art and science celebrities, prominent public figures, references to alcohol consumption as enhancing physical fitness, driving, mental performance, the solution to personal problems, referring to the stimulant, sedative effects or healing properties, associations with social success, higher libido or sexual performance. Advertisements promoting excessive consumption, higher strength as an advantage, negatively portraying abstinence and moderation were prohibited. Placement of advertisements was prohibited on the front and back pages of newspapers and their supplements, magazines, and books, specialized newspapers, magazines, books, television and radio programs for children and adolescents, from 3 p.m. to 10 p.m., and on weekends and school holidays, from 8 a.m. to 10 p.m. (excluding beer and wine with alcohol strength less than 15%), concerts, circuses, discotheques, and other mass events, theatrical performances, cinema and video screening sites such as education, science and education, all healthcare facilities, inside and outside public transport, gas stations, postcards, envelopes, and post stamps. Outdoor alcohol advertising was allowed, but a requirement to place a warning text about alcohol harms determined by the Ministry of Health was added. Alcohol advertising restrictions were controlled by the State Agency of Tobacco and Alcohol Control, while outdoor advertising was controlled by municipalities. 

Liberalization of advertising continued in 2002 [29]: the new amendments allowed advertising of alcohol below a volume strength of 22% and outdoor advertising was banned only for stronger beverages. Fines for all violations including advertising were reduced. In the next year, additional ad-liberalizing amendments [40] mandating new exceptions to the advertising ban were instituted. Display of alcoholic beverages was permitted in nearly all performing event halls (concerts, discotheques, theatre, cinema, etc.), and permitted in petrol stations. Trademark logos and names were excluded from the advertisement definition. 

A new restrictive attempt came into effect between 2007 and 2008 [41], which banned alcohol advertising on TV and radio during the daytime hours (6 a.m. to 11 p.m.). Restrictive measures came into effect approximately six months from the adoption. One more amendment [31] providing for a delayed full advertising ban from 2012 onward was adopted in 2008. Ultimately, on 6 December 2011, this amendment was revoked just before it could enter into force [32]. 

The next round of more restrictive alcohol advertising policy began in 2016 [42]. Amendment prohibited organizing games, actions, competitions, or lotteries to promote the purchase or use of alcohol, and alcohol was not to be used as a prize, gift, or bonus. Promotion of alcohol price reductions was prohibited. Then, in 2018, more than two decades after the first ban, a new comprehensive near-total ban on alcohol advertising [36] came into effect (including a full ban on TV, radio, and digital ads). Exempt from the ban was only the name and type of beverage, the name of the producer, the trademark (brand name), country of origin, geographical region of origin, ethanol content, labelling information, price in sales points, on producers and sellers’ websites as well as memorabilia. 

#### 3.1.4. Drink-Driving

There are 7 timeline points in the drink driving category. The control procedures were first defined with the adoption of the Law on Alcohol Control [16] and have become subject to administrative liability. In addition, it was permitted to forcefully transport the intoxicated person to medical services by the police if there was a risk of harm to others, an intoxicated person to themselves, and in the case of unconsciousness.

On 1 May 2003, changes in the Criminal Code came into effect [43] that introduced criminal liability when a drunk driver violated road traffic safety or their driving resulted in an accident causing an impairment to another person’s health or the victim suffered major property damage. 

On 1 January 2008, drink-driving legislation was toughened [44]. Penalties were increased, the option for car confiscation or driver imprisonment was introduced, and blood alcohol concentration (BAC) thresholds were reduced for young drivers from 0.4 to 0.2‰. A year later, starting in January 2009 [33], carrying alcohol in an opened package and consuming alcohol in cars were banned. 

Zero tolerance policy, introducing BAC 0‰, first came into effect on 1 January 2015 [45] and was applicable for professional drivers (taxi, buses, and trucks), novice drivers (under two years of experience), and all motorbike riders. 

From the beginning of 2017 [46], another amendment came into effect criminalizing drink-driving with a BAC level higher than 1.5‰. However, the amendment was flawed, since refusal to be tested by the police did not result in criminal offence. This was later amended and came into effect on 1 April 2019 [47]. 

#### 3.1.5. Taxation

There are 21 timeline points in the taxation category. Alcohol taxation in Lithuania began at the end of April 1994 after the Resolution On Excise Duties was adopted by the Parliament (a predecessor to the Law on Excise Duties), and the Government was granted responsibility for determining excise tariffs [48]. This continued for almost five years and excise tax was changed (mostly increased) six times [49,50,51,52,53,54] during that period. The amendments of the Law on Excise Duties was adopted on 1 January 1999 [55], and the excise rates were specified in the law. Hereafter, the excise tariffs could only be changed by amending the law, rather than by the Government decision. The power of decision over an effective control policy measure was moved from a policy implementation into a policy-making realm. Also in 1999, there has been a reduction of excise for some of the alcohol categories (ethyl alcohol and some fermented beverages) [56].

It is important to note that the form of excise tax has also changed throughout the years. In 1994, a tax was introduced as the specific amount charged per 1% of beverage alcohol strength or by value tax (a taxable value %, which adds a proportional tax to the value of alcoholic beverage excluding the excise tax and value-added tax). In 1995, both of these tax forms were combined for all alcohol product categories, with a safeguard for minimum taxation. However, a value tax was abolished in 1997 [54] when only a specific sum per 1% of actual alcoholic strength remained. This change may have potentially led to a reduction in excise tax when a proportional tax was removed from the price structure of more expensive types of alcohol. 

On 1 June 2001 [57], the excise tax was harmonized into a tax per hectoliter (hl) for ethyl alcohol, wines, and most of the intermediate products. Beer and mead brandy were included in this setup on 1 July 2002 [58]. The same amendment also produced an exemption: excise tax rate was reduced by half for small breweries (producing a maximum of 800,000 decaliters per year) on 100,000 decaliters sold per year.

Another excise tax reduction of 40% was applied to one category of fermented alcohol, which in 2004 was included in a broader category of intermediate products [59,60] as an attempt to balance tariffs for intermediate products. This resulted in four alcoholic beverage categories (ethyl alcohol, beer, fresh grape wine and other fermented beverages, intermediate products). These changes were part of legal harmonization with the EU law before joining the Union. Licensing (except for agricultural ethyl alcohol and wines) was also removed for imports and exports from the EU and the beverage volume strength requirements were adjusted to the EU regulations [60]. 

After Lithuania joined the EU in 2004, the excise tax increased on another eight occasions [61,62,63,64,65,66,67,68]. The most significant excise tax increase was on 1 March 2017 [66] when the excise for beer and wine more than doubled, while that for the intermediate products increased by more than 90%, and for ethyl alcohol by 23%. The more detailed summary of the excise duty changes is provided in Supplementary Materials Table S1.

### 3.2. Brief Examples of the Policy Development Process

#### 3.2.1. Example 1: Alcohol Sale Ban in Petrol Stations Which Came into Effect in 2016

The amendment for the ban on alcohol sales in petrol stations was adopted in 2011 after a proposal by 25 MPs for entry into force in January 2016. In March 2015, in anticipation of the upcoming ban, three new amendments of the Alcohol Control Law were registered: one to postpone the ban until the beginning of 2019, another to ban only the sale of alcoholic beverages stronger than 15% from 2018, and one comprehensive proposal covering a broad range of alcohol control measures.

The representatives of petrol stations rigorously opposed the ban, claiming that they adhered to all official regulations and the decision would result in job losses for a third of their workforce [69]. To test the claims that petrol stations abided by the age limit in alcohol sales, in April 2015, three civil society activists conducted a mystery shopping experiment [70] that demonstrated that an underage high school student was able to purchase alcohol in many petrol stations without validating their age. The case attracted much public attention, and some of the businesses who were given fines later sued the Department for Drug, Tobacco and Alcohol Control, insinuating that the adolescent had “provoked a crime” [71].

In September, a young policewoman was fatally injured by the drunk truck driver, and the Government pledged support for the amendments coming into effect on time. Opaque manoeuvring in the parliamentary committees increased after that in parallel with an extensive public discussion on the issue. At the very beginning of November, four women were murdered by an intoxicated and alcohol-addicted man, which again increased support among the public for tougher alcohol control. In the last two months of the year, NGOs produced relentless pressure through publicity, articles, interviews, producing a timeline of the process [14] and enlisting the support of international organizations (Eurocare, NordAN, etc.) in supporting the ban. Meanwhile, intensive negotiations were taking place in the parliamentary committees with additional suggestions, confusing amendments, and new proposals to delay the coming into effect dates. Between September and November, five health committee meetings were held and at least four additional amendments or improved wordings was discussed and voted in the Committee for Health Affairs.

On December 1, yet another amendment was unexpectedly registered for the discussion in the parliamentary session agenda that aimed to revoke the ban, authored by the same MP who originally proposed the introduction of the ban in 2011. On the same day, the proposal was removed from the agenda, only to return and be discussed on 2 December. On the next day, a standing rally was carried out inside the Parliament building by the employees of petrol stations claiming that they would lose their jobs if the ban came into effect [72]. It also became public knowledge that all of the MPs who authored the newest amendment against the ban revoked their signatures. However, one MP managed to sign “in the moments between” of all the signatures being revoked and the proposal lost its status as an amendment, and therefore the issue was kept on the agenda. The ‘war’ ensued between efforts to place the issue up for voting in the agenda and to take it out, using the procedural knowledge of the parliamentary processes. 

On December 12, an adolescent was killed by a drunk driver. On December 15, employment reports demonstrated that in 2015, the number of petrol station employees was growing. On December 17, the amendment to revoke was still not on the agenda for the last parliamentary session before the break, but the Committee of Health Affairs produced a request for the Ministry of Energy to update and improve the definition of a “petrol station”. On December 22, it became public that there was an additional attempt to add the revoking amendment up for voting during the last parliamentary meeting before the winter break. It lost only by two votes. On December 23, the last meeting of the Parliament in 2015 was held without discussing a revoking of the ban. On 1 January 2016, the alcohol sales ban in petrol stations came into effect. On 6 January 2016, one of the retail chains of petrol stations announced that they would discontinue services at night. This amendment reduced the accessibility of alcohol by 600 retail spots and the claim about massive unemployment in petrol stations turned out to be false [73].

#### 3.2.2. Example 2: A Brief Industry Campaign of Universal Personal ID Verification for Buying Alcohol in 2016

On 30 June 2016, the Association of Lithuanian Trade Enterprises, the seven largest Lithuanian retail chains and the Ministry of Health signed a memorandum of goodwill [74]. According to the memorandum starting on 2 September 2016, retailers of alcoholic beverages voluntarily began to request an ID from all buyers of alcoholic beverages, despite their age. The retailers maintained that as a socially responsible industry, they would offer voluntary and effective solutions that were superior to the provisions of the Alcohol Control Law. It was also hoped by the interest groups that this might eliminate the need for the proposed increase in the legal purchasing age. The initiative caused much public controversy, though interestingly, in the public perception, the Parliament was the main culprit of the inconvenience customers experienced in buying alcohol, even though the initiative came from the private sector [75].

The Parliament reacted very quickly by amending the Law on Alcohol Control that came into effect on 8 October 2016 [76], with the amendment stating that sellers of alcoholic beverages could only request a customer purchasing alcoholic beverages to present a valid ID, if there was doubt that the person was younger than 18.

The case reflects the tradition of alcohol control policy being based on laws and therefore the possibility of subversion, since every control measure is perceived in the society as if it is instituted by the highest authority of the state and therefore legal. Among the public, the responsibility of a controversial decision was attributed to the Parliament, while it may have been a voluntary action by the retailers potentially aimed at discouraging the proposed new amendments of the legal age limit.

#### 3.2.3. Example 3: Journal Page Ripping Initiative: A Response to the Advertising Ban in 2018

Since January 2018, a near-total alcohol advertising ban came into effect in Lithuania that applied to all of the media and retailers. All of the alcohol advertising has since disappeared from TV, radio, digital media, and printed media in Lithuanian language (including foreign publishers targeting printing in the national language). In general, the industry has complied with the new restrictions with one interesting special exception: importers of foreign magazines printed in a foreign language. Even though print foreign journals account for only for a small fraction of the media market, their response has resulted in a major media outburst and increased visibility of negative opinions in the society toward the overall ban of alcohol advertising [77].

Once the ban came into effect, the distributors of imported journals started to remove pages or put stickers on pages with alcohol ads, claiming that this was required by law. Even though between adoption and coming into effect of the law there was a period of half a year, which could have been used to prepare for the implementation, the problem was vigorously flagged by the distributors only immediately after the law came into effect. 

The Ministry of Economy prepared a draft of the Law on Alcohol Control with the exception for alcohol advertising in foreign printed mass media; this project was registered in the Parliament legal documents database, but the procedure for parliamentary discussion was not initiated. Lithuanian Tobacco and Alcohol Control Coalition has held an international consultation within the NordAN network and issued a recommendation for the government to not change the law, but to continue with less formal practices of control. The hostile actions of the distributors ended, the legislation was not changed, and the issue seems to have lost all significance. 

## 4. Discussion

The main limitations of the study are that only the perspectives of public health and not commercial vested interests were analyzed. The analysis did not cover legislative changes related to the industry or economic issues, with less detailed focus on licensing, production regulation, etc. Additionally, it did not cover anything other than the five national policy areas that were outlined in the Global Strategy. Although other policy areas were ignored in this analysis as having less impact on population alcohol consumption, they do influence specific target groups such as persons affected by harmful drinking or addiction. Further analysis could also investigate in more detail how political decisions have affected these particular groups.

Ideological and industry influences were beyond the scope of this study and require additional research. Influence of the lobby groups is extremely difficult to elucidate once the decisions are made because there is often no recorded evidence of pressure from interest groups. Therefore, the brief examples analyzed in this study brings added value to the understanding of the complexity of legislative and political processes and how they might be influenced by lobbyists or public opinion. 

Regaining independence created a vacuum of regulations pertaining to alcohol control for several years. This period coincided with the increase in alcohol consumption and the rapid decline in life expectancy [8]. 

The period of 1994–1997 can be referred to as the beginning of alcohol control policy in Lithuania: it included the formation of alcohol control policy, the introduction of alcohol taxation, regulation of availability, restriction of advertising, and establishing relevant control institutions. From then on, the policy process went through several cycles of stricter control and liberalization: intensifying control policies (1994–1997; 2008–2009; 2014–2020) and mitigation of control (1990–1994; 1998–2007). 

The frequent changes to the main policy document, The Law on Alcohol Control, might be the evidence of the intensity of the political struggle between public health and industry lobby interests. The Parliamentary process is crucial for the legal transformation of alcohol control policy in Lithuania. This is reflected in the efforts of NGOs to evaluate the effect of Members of Parliament (MPs) voting patterns on public health. For instance, analysis of the voting patterns of MPs from 2012 to 2018 revealed 95 voting episodes amending the Law on Alcohol Control and the Law on Excise Duties [78]. Every political party has a clear position regarding the alcohol policy measures they favour. This contributes to tension, resulting in heightened legislative activity in this area. It is not surprising that due to constantly changing political leadership, there have been many amendments to legal documents. 

However, despite the political changes during the past decade, Lithuania has managed to adopt comprehensive alcohol control policies. The period from 2007 to 2020 marks the implementation of so-called ‘best buys’ and coincides with a significant increase in male life expectancy [79]. There is an increased interest in the effects of Lithuanian alcohol control policies toward specific health indicators, and studies have already showcased positive health results [80].

In recent years, there has been significantly greater involvement of non-governmental organizations and the public in political processes. The most noteworthy was the campaign “For Sober Lithuania”, which collected more than 60,000 signatures to advance evidence-based alcohol control policies [81].

There have been other events aside from legislative processes that might have had a strong impact on population alcohol consumption. Among such significant events were the Russian economic crisis of 1998, Lithuania joining the EU in 2004, the global economic crisis in 2008–2010, and the recent COVID-19 pandemic and crisis. Additionally, with the free movement of goods and citizens across the EU, cross-border trade of alcohol products has had a noticeable impact on national policies. Taxation strategies, in particular, have been at the center of discussions varying from more harmonized taxes in comparison with their neighbours to an increase in excise duty.

Among the comprehensive legislative measures that are important for alcohol control, the national health programs have to be mentioned. These are strategic public health documents that specifically mention targets for population alcohol consumption. On 2 July 1998, the Parliament adopted the resolution regarding the first Lithuanian health program for the period of 1998–2010. This program included a specific target of reducing alcohol consumption by 25 per cent, also reducing the prevalence of alcohol psychosis. It also listed specific means for achieving these goals, which included price increases and potential alcohol sale monopolies, among others [18]. Achieving the targets outlined in the National Health Program is the central focus of the Lithuanian Law on Health. On 26 June 2014, the Parliament revised the program (renamed the Lithuanian Health Strategy in 2016) [22]. This strategy established specific population consumption targets of legal alcohol for 2020 and 2025 as 9.5 and 8.5 liters, respectively. 

An important role was played by the Lithuanian Constitutional Court, which since 1997 had ruled that alcohol and tobacco were ‘special purpose goods’ and that restrictive policies toward these products may be applied in Lithuania in order to ensure the health of the citizens. 

From 2010 to 2015, Lithuania was listed among the top drinking countries globally, which sparked a lot of public debate and increased pressure toward legislative action. It eventually translated into the adoption of evidence-based policies from 2016. When Lithuania began to implement stricter policies back in 2018, the World Health Organization claimed that inaction on alcohol control on a global level was widespread. However, it was acknowledged that political commitment at the highest level to implement effective interventions had contributed substantially to the sharp reduction in alcohol use and related harm in Eastern Europe. [1] 

There is no doubt that alcohol control policy is extremely complex and spans over many fields such as legal environment, healthcare, economy, social affairs, and many others. Therefore, this study could be useful not only for the assessment of the development of alcohol control legislation in Lithuania, but also serve as a background summary for the future international comparative studies, once the impact of Lithuanian alcohol control policies is assessed [80,82]. The timeline helps to establish benchmark moments of impact for the WHO’s ‘best buy’ interventions (taxation, alcohol advertising ban, measures to reduce availability) implemented in Lithuania during a short period. 

## 5. Conclusions

The development of alcohol control policy in Lithuania reflects the complexity confronting decision makers in balancing the economic and public health interests. The Lithuanian national alcohol control policy is centered on laws, rather than expert derived policy documents. During the past three decades, the policy has undergone several cycles of stricter control and liberalization, resulting in numerous legislative amendments. Transparent democratic parliamentary processes are crucial to success in protecting public health, especially when the processes are reinforced by the active involvement and support of civil society. Some of the recent periods when a series of ‘best buy’ interventions were implemented have produced a basis for a natural experiment and a need for future studies of policy impact.

## Supplementary Materials

The following are available online at https://www.mdpi.com/1660-4601/17/10/3454/s1, Table S1: Changes in alcohol excise tax in Lithuania 1990-2020.
